# The Early Growth of Maize Under Waterlogging Stress, as Measured by Growth, Biochemical, and Molecular Characteristics

**DOI:** 10.3390/biology14020111

**Published:** 2025-01-17

**Authors:** Ana Nikolić, Manja Božić, Nikola Delić, Ksenija Marković, Marija Milivojević, Zoran Čamdžija, Dragana Ignjatović Micić

**Affiliations:** 1 Maize Research Institute “Zemun Polje”, Slobodana Bajića 1, 11185 Belgrade, Serbia; mbozic@mrizp.rs (M.B.); kmarkovic@mrizp.rs (K.M.); mmarija@mrizp.rs (M.M.); zcamdzija@mrizp.rs (Z.Č.); idragana@mrizp.rs (D.I.M.); 2 Faculty of Biology, University of Belgrade, Studentski trg 16, 11158 Belgrade, Serbia; nikdelic@gmail.com

**Keywords:** maize, climate change, waterlogging, ROS, photosynthesis

## Abstract

An effective strategy to address the impacts of climate change on maize involves early planting, which mitigates drought stress during critical growth phases, preventing yield reductions. The research assessed two maize inbred lines (sensitive and tolerant to low temperature) under conditions of waterlogging stress. This is crucial since early sowing often faces both low temperatures and heavy rain. Morphological, biochemical, and molecular responses were recorded after 24 h, 72 h, and 7 days of stress during the growth stage of 5-day-old seedlings. The findings indicated a more pronounced decline in all morphological characteristics in the sensitive line. Both genotypes displayed an increased root-to-shoot ratio, suggesting that the shoots deteriorate more rapidly than the roots. Physiological evaluations demonstrated that the tolerant line was more effective in managing ROS levels compared to the sensitive line. The involvement of H_2_O_2_ in aerenchyma formation implies that the decreased POD activity and elevated MDA levels observed after seven days may be associated with aerenchyma development in the tolerant line. Genes essential for PSII function revealed that waterlogging adversely affected photosynthesis in the sensitive genotype. In summary, the low-temperature tolerant genotype exhibited significant resilience to waterlogging, indicating potential interaction between the pathways governing these two abiotic stressors.

## Simple Summary

Climate change negatively affects the growth of important crops worldwide, including maize. To address this issue and reduce the harmful effects on crop yields, the agricultural sector is creating various strategies. One effective approach is early planting to reduce drought stress during critical growth phases, helping to avoid yield losses. This research evaluated how two maize inbred lines, one sensitive and one tolerant to cold respond to waterlogging stress. This is significant because early planting often occurs during cold weather and heavy rainfall. The research looked at the physiological and genetic responses during early growth stages. The results indicate that the cold-tolerant line also shows good tolerance to waterlogging, suggesting that similar mechanisms may be at play in how plants respond to both stresses, raising questions about their ability to manage both cold and excess water.

## 1. Introduction

The effects of climate change and global warming are projected to cause a substantial increase in precipitation and flooding worldwide, with Europe being no exception. Such phenomena are expected to adversely impact crop yields [1]. The rise in the frequency of heavy precipitation events results in waterlogging stress, with the duration of this stress being affected by factors such as rainfall intensity, drainage conditions, and soil structure [2].

Unfavorable effects arise during prolonged periods of waterlogging (weeks) as well as in short-term scenarios that occur over hours or days [3]. Detrimental impacts are also influenced by various factors, including species, genotype, environmental conditions, duration of the stress, and growth stage.

Severe damage caused by waterlogging have been detected in most important crops worldwide: wheat [4], rice [5] and maize [6]. Even rice, which is adapted to endure shallow flooding (under 5 cm) and is thus waterlogging tolerant, can still suffer from significant stress after heavy summer rains due to the prolonged presence of water. Maize, not being a wetland crop species, is highly prone to the adverse effects of excess moisture and waterlogging stress [7].

The extent of waterlogging duration was recognized as an essential factor affecting plant survival, including maize [8]. Evidence from different studies indicated that as the length of stress exposure increased, crop production experienced a decline [9,10,11].

Another significant factor to consider is the growth stage of maize affected by stress. Studies demonstrated that maize experiences sensitivity to waterlogging stress especially during the third leaf stage (V3), the sixth leaf stage (V6), and tasseling (VT) [11,12].

Due to waterlogging stress, reduced oxygen availability occurs in the plant root area, creating hypoxic or anoxic environment. These detrimental conditions provoke a series of changes at the morphological, anatomical, physiological, biochemical, and molecular levels.

The presence of these conditions obstructs root respiration, negatively affecting the rates of photosynthesis and the assimilation of carbon dioxide. The reduction in root conductance limits the absorption of water and nutrients, ultimately resulting in inadequate plant growth [2]. Different studies indicate that plants possess the ability to modify their growth strategies, reducing vertical development while reallocating resources towards lateral growth to enhance optimal nutrient absorption [13]. At the anatomical level, various alterations in root structure occur, including the formation of adventitious roots and the development of aerenchyma [14]. In contrast to fostering the growth of specialized root structures, waterlogging can restrict root development and lead to the death of root segments as a result of oxygen deprivation. The limitation of root growth can significantly impair the growth and development of plants, given that it limits their access to necessary nutrients [15].

An obstruction in the exchange of oxygen disturbs the physiological and biochemical functions within plants. The adverse impacts of waterlogging stress on the photosynthetic apparatus of plants result in an excessive generation of reactive oxygen species (ROS). Reactive oxygen species (ROS) damage molecular and cellular components and induce lipid peroxidation that leads to the impairment of cellular membranes, enzymes, nucleic acids, and proteins, ultimately culminating in cell death [16]. To avert harm to cellular structures, the antioxidant defense system is activated, which consists of several enzymatic antioxidants. Enzymatic antioxidants include a diverse array of enzymes, such as peroxidase (POD), catalase (CAT), superoxide dismutase (SOD), ascorbate peroxidase (APX), dehydroascorbate reductase (DHAR), monodehydroascorbic acid reductase (MDHAR), and glutathione reductase (GR).

Beyond the other structures and processes that impacted the effects of waterlogging stress, there is a concurrent alteration in the expression of many waterlogging responsive genes involved in signal transduction as well as transcriptional and translational regulation [6]. A wide array of studies established that specific genes are significantly affected by waterlogging stress in plant organisms [17,18]. The stress caused by waterlogging affects the expression of genes that are significantly associated with the shift of plants towards anaerobic metabolism. In environments lacking oxygen, a notable increase in PDC (*Pyruvate decarboxylase*) gene family activity was thoroughly recorded in plant species such as rice, wheat, and maize [19]. As indicated earlier, oxidative stress results in a sequence of changes at different levels, including the anatomical alteration of roots, characterized by the formation of aerenchyma. The development of aerenchyma involves modifications to the cell wall, regulated by the mechanisms of loosening and expansion [20]. The degradation of the cell wall occurs through the synergistic activity of pectolytic, xylanolytic, and cellulosolytic enzymes [21], which is indicated by the increase in the expression of their respective genes. Moreover, different abiotic stresses modulate the activity and expression of ATP-sulfurylase in plants via different S-compounds [22]. In the context of hypoxia or anoxia, photosynthesis frequently represents one of the primary systems impacted. Consequently, it is to be expected that the expression of genes associated with the components of the photosynthetic apparatus may also undergo modifications in plants [23].

The focus of this study was to evaluate the responses to waterlogging stress in two maize lines (one sensitive and one tolerant to low temperature). These genotypes were selected to be evaluated for waterlogging tolerance since both periods of suboptimal temperatures and heavy rainfall are characteristic for the time of year when early sowing would occur. A possible overlap in the underlying mechanisms raises a question how similar the physiological or biochemical pathways are in plants used to cope with both stresses. As early sowing is a promising strategy that could mitigate adverse effects of drought stress during the flowering and grain filling periods and thus ensure sustainable yield, understanding the underlying mechanisms is of the utmost importance.

The experiment was carried out under both controlled and waterlogging stress conditions, with the objective of elucidating the mechanisms that contribute to adaptations or resistance to stress during very early developmental stages of maize. The research focused on the morphological, physiological, and molecular responses of one tolerant and one susceptible maize line to low temperature stress, in order to test the congruence of tolerance to two different stresses. Also, three specific periods of stress exposure were applied to evaluate the effects of stress duration.

## 2. Materials and Methods

### 2.1. Plant Material and Experimental Design

Previous research [24] identified two maize genotypes of contrasting tolerance to low-temperature stress: L_T_, considered tolerant, and L_S_, considered susceptible to low temperatures. L_T_ belonged to the BSS and L_S_ to Lancaster heterotic group. The assessment of tolerance to low-temperature conditions of these genotypes was based on seed vigor and survival rate, as well as morphological parameters, including radicle length, coleoptile length, and seedling fresh weight.

Sixty seeds of booth per genotype were sterilized in 1% sodium hypochlorite solution (commercial bleach) and germinated in a growth chamber (MLR-352H-PE, PHC Europe B.V.) for five days. The germination was performed in the dark for the first five days, under optimal conditions (30/20 °C; 75% relative humidity, RH). The 5-day-old seedlings were transferred into custom-made hydroponic pots containing a standardized plant nutrient solution developed by Knop: Ca(NO_3_)_2_ (1000 mg/L), MgSO_4_ (250 mg/L), KH_2_PO_4_ (250 mg/L), KNO_3_ (250 mg/L), and FeSO_4_ (20 mg/L). The pots had partitions with holes, placed 10 cm below the top edge, through which roots were submerged into the Knop solution. The pots were returned to the growth chamber (30/20 °C; 8/16 h, 75% relative humidity, RH) exposed to waterlogging stress (T) by submerging them fully into the Knop solution—the solution level was 2 cm above the tallest seedling. The sampling was performed after 24 h, 72 h, and seven days of stress treatment. Morphological parameters were measured on 10 plants per replicate immediately after sampling, while the samples of the same size for enzyme activity, lipid peroxidation, and gene expression analysis were ground in liquid nitrogen and stored at −80 °C for further research. The analyses were carried out in three separate replicates, with each genotype represented by ten maize plants. Control plants (Cs) were grown under optimal conditions in the same period and sampled at identical time points—the shoots were fully above the level of the Knop solution.

### 2.2. Growth Parameter Assessment

Morphological parameters analyzed in this study included the fresh weight (FW), radicle length (Lrad), and coleoptile length (Lcol) for plants sampled after 24 h and 72 h of stress treatments. For plants exposed to 7 d of anaerobic conditions, root (RFW) and shoot (SFW) fresh weight, as well as root (RL) and shoot (SL) length, were measured immediately after sampling. Additionally, dry root (RDW) and shoot (SDW) weights were determined after the plants were dehydrated for 24 h at 110 °C in a drying oven, and root/shoot ratios (R/S) were calculated according to the formula: R/S = RDW/SDW. Finally, the survival rates of both genotypes were calculated as the percentages of plants surviving stress treatment after 7 d.

### 2.3. Physiological Parameter Assessment

#### 2.3.1. Measurement of Lipid Peroxidation

Lipid peroxidation levels were measured indirectly by determining the concentration levels of malondialdehyde (MDA), a compound formed by lipid peroxidation and plasma membrane decomposition, according to Heath and Packer (1968). Briefly, 0.1 g plant tissue was dissolved in 0.1% trichloroacetic acid (TCA). After incubating (20 min at 4 °C) and centrifuging the samples (12,000× *g*), 500 μL of supernatant was separated and combined with 1.5 mL of 0.5% thiobarbituric acid (TBA) dissolved in 20% TCA. The samples were then incubated at 95 °C for 40 min, centrifuged at 12,000× *g* for 15 min (4 °C), and the supernatant was used to record the absorbance values at 532 nm and 600 nm (BioSpectrometer, Eppendorf, Hamburg, Germany). The measured absorbance values (A) were used to determine the MDA concentration in the samples according to the formula:

$$MDA\left(\frac{nmoL}{g_{FW}}\right)=\frac{V\times f\times (A_{532}-A_{600})\times 1000}{\varepsilon }\times m$$

V is the reaction volume (V = 0.5 mL), f is the dilution factor (f = 2), ε is the extinction coefficient of MDA at 532 nm (ɛ = 155 mM^−1^cm^−1^), and m is the plant tissue mass (m = 0.1 g).

#### 2.3.2. Antioxidative Enzyme Activity Analysis

Enzymes included in this analysis were peroxidase (POD) and ascorbate peroxidase (APX), and their activities were measured based on the protein concentration (c_prot_) in the sample, determined through Bradford’s test [25]. The standard calibration curve was fitted based on the known dilutions of bovine serum albumin (BSA). In brief, 20 μL of BSA standard solutions (1000 μg/mL, 500 μg/mL, 400 μg/mL, 250 μg/mL, 100 μg/mL, 50 μg/mL, 25 μg/mL, and 0 μg/mL) were dissolved in 2 mL of 1× Bradford’s solution. The absorbance of each of these solutions was measured at 596 nm, and the standard curve was fitted.

POD activity was determined by measuring the absorbance at 420 nm of pyrogallol quinone, a compound formed by the oxidation of pyrogallic acid, catalyzed by POD. In alignment with the manufacturer’s instructions, the peroxidase (POD) Activity Assay Kit (Elabscience^®^, Wuhan, China) was employed for this purpose. The enzyme amount that can catalyze the oxidation of 1 μg of in 1 mg protein per minute at 37 °C was defined as one unit. The POD activity was calculated according to the following formula:

$$POD(U/{mg}_{prot})=\frac{\frac{A_{sample}\;-\;A_{control}}{12\;\times \;OP}\times \frac{V_{1}}{V_{2}}}{1000\times t\times (c_{prot}-f)}$$

OP is the optical pathway, or the cuvette width, V_1_ is the total reaction volume, V_2_ is the sample volume, t is the reaction time, c_pr_ is the protein concentration (mg_prot_/mL), and *f* is the dilution factor.

To evaluate the activity of APX, the reduction in ascorbic acid (ASA) levels was analyzed, given that APX catalyzes the transformation of ASA into monodehydro-l-ascorbic acid (MDHA). This evaluation was conducted by measuring the decrease in absorbance at 290 nm, employing the ascorbate peroxidase (APX) Activity Assay Kit (Elabscience^®^) as per the manufacturer’s instructions. One unit of APX was determined as the amount of enzyme that catalyzed 1 μmol of ASA in 1 mg of protein, in 1 mL reaction system per minute, according to the following formula:

$$APX\;\left(U/{mg}_{prot}\right)=\frac{\frac{\Delta A}{\varepsilon \;\times \;OP}}{t}\times \frac{V_{1}}{V_{2}\times c_{prot}}\times f$$

ΔA = A_1_ − A_2_ (A_1_—initial absorbance at 290 nm, A_2_—apsorbance after 2 min of reaction), OP is the optical pathway, or the cuvette width, ε is the extinction coefficient of ASA at 290 nm, OP = 1 cm (2.8 mL/μmoL × cm), t is the reaction time, V_1_ is the total reaction volume, V_2_ is the sample volume, c_prot_ is the protein concentration (mgprot/mL), and f is the dilution factor.

### 2.4. Gene Expression Assays

Gene expression analysis was performed by comparing the expression levels of genes important for waterlogging and abiotic stress. This comparison was made between the control and treatment samples of both genotypes across all three time points, employing real-time PCR. The genes included were as follows: pyruvate decarboxylase 1 (*Zmpdc1*), pyruvate decarboxylase 3 (*Zmpdc3*), xyloglucan endotransglycosylase (*XET*), chloroplast ATP-sulfurylase (*ATPS*), photosystem II reaction center protein I (*psbI*), and photosystem II cytochrome b559 alpha subunit (*psbE*).

Firstly, total RNA was extracted using 100 mg of the frozen plant tissue and Gene Jet RNA Purification kit (Thermo Scientific™, Waltham, MA, USA). Possible DNA contamination was eradicated from the extracted RNA samples using DNase I (Ambion^®^ DNA-free™ kit, Invitrogen™, Waltham, MA, USA). Total RNA concentrations were determined by the NanoDrop™ spectrophotometer (Thermo Scientific™), and the RNA quality, integrity, and degradation were further assessed through agarose gel electrophoresis. Complementary DNA (cDNA) was synthesized from 1 μg of the purified, total RNA, by implementing the Revert Aid First Strand cDNA synthesis kit with RNase inhibitor (Thermo Scientific™). The cDNA synthesis was confirmed through agarose gel electrophoresis, using 26S primers.

Real-time PCR reactions were performed in reaction mixtures (10 μL) containing 1x HOT FIREPol^®^ EvaGreen^®^ qPCR Mix Plus (ROX) (Solis BioDyne™, Tartu, Estonia), 0.2 μM of forward and reverse primers and 1 μL of template cDNA diluted 2 times, on a StepOnePlus™ Real-Time PCR System (Applied Biosystems™, Waltham, MA, USA). PCR amplification included 40 cycles of denaturation (95 °C for 15 s), primer annealing, and extension (annealing temperature, T_A_, appropriate for each primer for 60 s). Cyclophilin (*cyp*) was used as the internal reference gene [26]. Primers for the selected genes were taken from [27] (*Zmpdc1*, *Zmpdc3*), (*XET*), [28,29] (*ATPS*, *psbI*, *psbE*). All the primers used are listed in Table 1. The appropriate amplification efficiencies were calculated according to E = 10^(−1/slope)^ method. Relative gene expression was calculated according to [30] applying efficiency correction as in [31].

### 2.5. Statistical Analysis

The statistical analyses were completed using the stats package in R (v4.3.3) [32]. The distribution of data obtained from all of the analyses was checked for normality using the Shapiro–Wilk and Kolmogorov–Smirnov test. Student’s *t*-test was carried out for mean comparison with a significance level at *p* < 0.05 for all analyses (morphological parameters, lipid peroxidation, POD and APX activity and gene expression analysis). Graphs were designed in Microsoft Excel (Windows 10).

## 3. Results

### 3.1. Analysis of Morphological Traits for Assessing Tolerance to Waterlogging

Several morphological parameters were taken into consideration when assessing the waterlogging tolerance of the two genotypes: FW, Lrad, and Lcol (for plants sampled after 24 h and 72 h) and RFW, SFW, DRW, DSW, RL and SL (for plants sampled after 7 d). The results indicate that both genotypes performed comparably under anaerobic conditions after 24 h; however, they exhibited differences following extended exposure to waterlogging.

**Figure 1:**
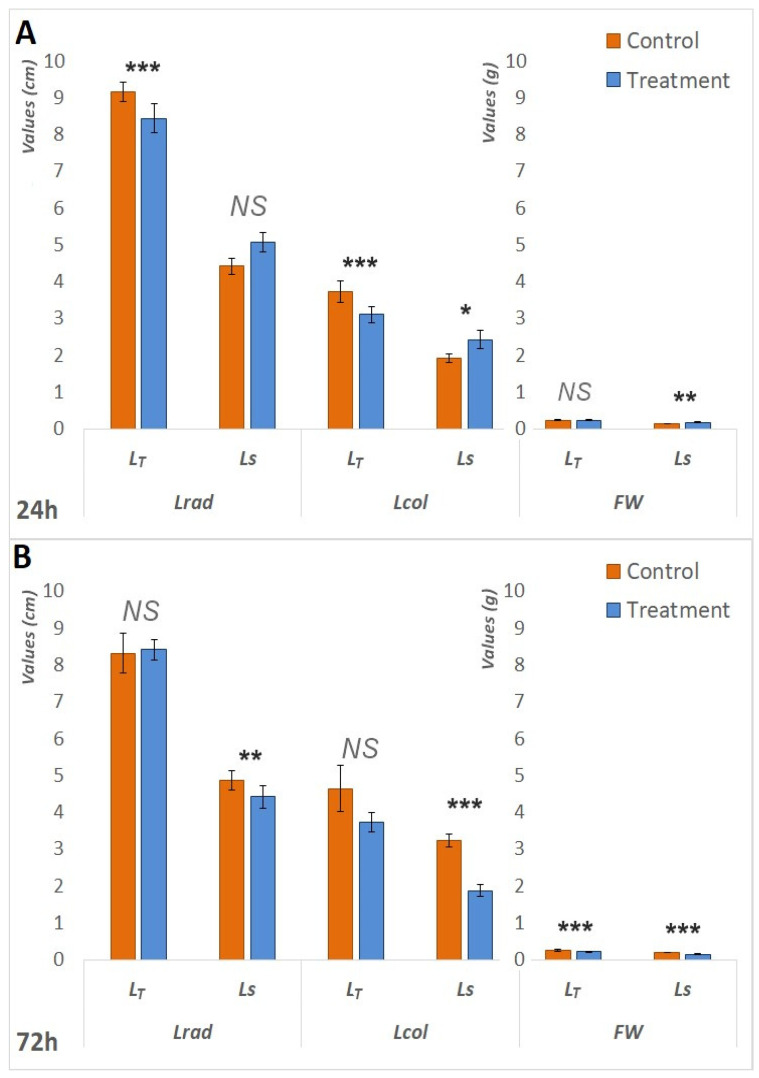
Differences in measured morphological parameters between the control and treatment after 24 h and 72 h. (**A**). Differences in Lrad, Lcol, and FW between the control and treatment of both L_T_ and L_S_ after 24 h of waterlogging treatment. (**B**). Differences in Lrad, Lcol, and FW between the control and treatment of both L_T_ and L_S_ after 72 h of waterlogging treatment. Measured parameters include radicle length (Lrad), coleopatile length (Lcol) measured in centimeters (cm), and seedling fresh weight (FW) measured in grams (g). The significance of the difference between the control and treatment of each parameter was determined by *t*-test and is shown as *** (*p* < 0.001), ** (*p* < 0.01), * (*p* < 0.05), and NS (statistically not significant at *p* > 0.05).

**Figure 2:**
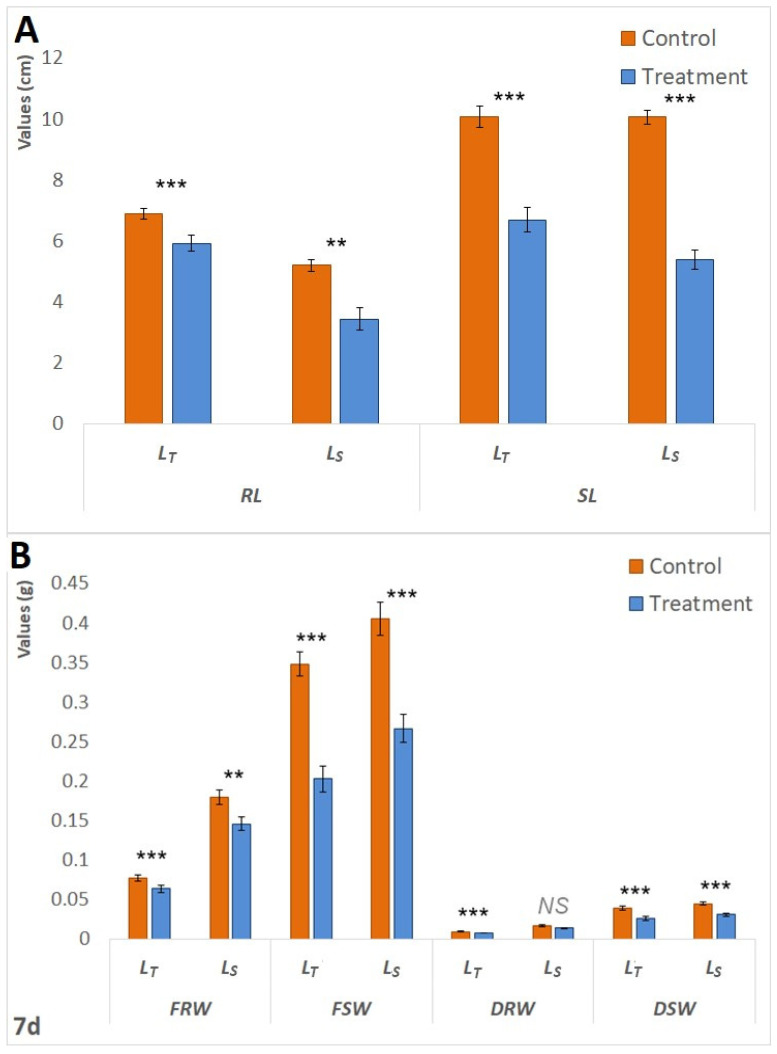
Differences in measured morphological parameters between the control and treatment after 7 d. (**A**). Differences in RL and SL between the control and treatment of both L_T_ and L_S_ after 7 d of waterlogging treatment. (**B**). Differences in FRW, FSW, DRW, and DSW between the control and treatment of both L_T_ and L_S_ after 7 d of waterlogging treatment. Measured parameters include root langth (RL), shoot length (SL) measured in centimeters (cm), root (RFW) and shoot fresh weight (SFW), and root (DRW) and shoot dry weight (DSW) measured in grams (g). The significance of the difference between the control and treatment of each parameter was determined by *t*-test and is shown as *** (*p* < 0.001), ** (*p* < 0.01), and NS (statistically not significant at *p* > 0.05).

Initially, following 24 h of waterlogging treatment, L_S_ exhibited superior performance compared to L_T_. Statistically significant increases in Lcol (*p* < 0.05) and FW (*p* < 0.05) were observed in the treated seedlings of L_S_. In the context of L_T_, the comparison between the control and treatment groups demonstrated statistically significant decreases in both Lrad (*p* < 0.001) and Lcol (*p* < 0.001), as illustrated in Figure 1.A. However, as the waterlogging period extended, L_S_ became more affected. Following the 72 h treatment period, Figure 1B). Furthermore, both genotypes exhibited a statistically significant (*p* < 0.001) reduction in fresh weight (FW) in treated plants after 72 h; however, the reduction was more pronounced in L_S_. Specifically, the FW in treated L_S_ plants was 25% lower than that of the control seedlings, whereas the decrease in L_T_ was 15%. The same pattern was observed after seven days of treatment. The waterlogging had a negative impact on the fresh and dry root and shoot weights of both genotypes (Figure 2B), but RL and SL showed a less pronounced difference in L_T_ (Figure 2A). The survival rates of L_T_ seedlings were significantly superior to those of L_S_ seedlings; 100% of L_T_ seedlings endured the 7-day treatment, whereas one-third of the L_S_ plants did not survive.

### 3.2. Analysis of Physiological Traits for Assessing Tolerance to Waterlogging

The study of waterlogging tolerance included the levels of lipid peroxidation and the activity of antioxidative enzymes peroxidase (POD) and ascorbate peroxidase (APX).

**Figure 3:**
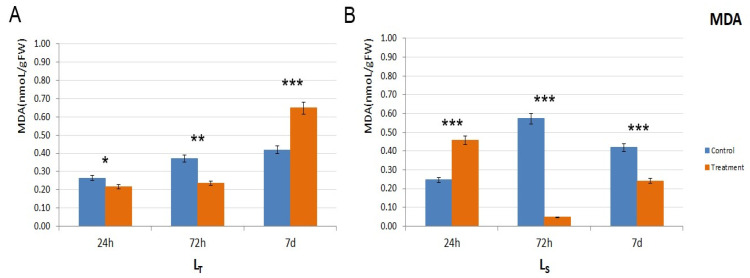
MDA levels in the control and treatment samples of both genotypes. (**A**). MDA levels in L_T_, in all three time points (24 h, 72 h, and 7 d) in control (shown in orange) and treatment conditions (shown in blue). (**B**). MDA levels in L_S_, in all three time points (24 h, 72 h, and 7 d) in control (shown in orange) and treatment conditions (shown in blue). MDA level is shown as nanomols per gram of fresh tissue (nmol/gFW). The significance of the difference between the control and treatment of each parameter was determined by *t*-test and is shown as *** (*p* < 0.001), ** (*p* < 0.01), * (*p* < 0.05).

MDA concentration was measured to ascertain the extent of lipid peroxidation. In L_T_, the MDA levels were higher in the control conditions after 24 h and 72 h, but a threefold increase was noted in the stressed plants after 7 d, which was 50% higher compared to the control (Figure 3A). However, in L_S_, the situation was different in L_S_: after the initial peak after 24 h (app. double the control value) of waterlogging conditions, MDA levels declined after 72 h and 7 d and were lower than the control levels (Figure 3B).

**Figure 4:**
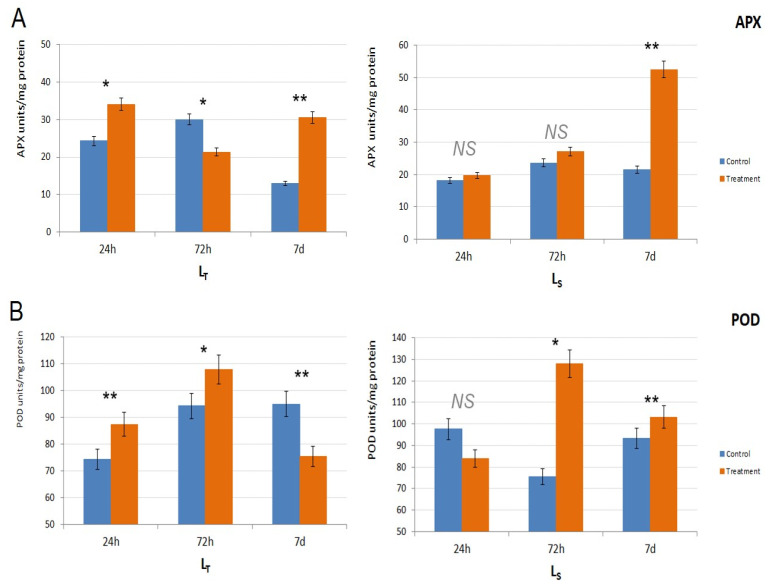
Antioxidative enzyme activity in the two genotypes. (**A**). Ascorbate peroxidase (APX) activity in L_T_ and L_S_, in all three time points (24 h, 72 h, 7 d). APX activity is shown as APX units/mg of protein. (**B**). Peroxidase (POD) activity in L_T_ and L_S_, in all three time points (24 h, 72 h, 7 d). POD activity is shown as POD units/mg of protein. Control samples are shown in orange, and treatment samples are shown in blue. The significance of the difference between the control and treatment of each parameter was determined by *t*-test and is shown as ** (*p* < 0.01), * (*p* < 0.05), and NS (statistically not significant at *p* > 0.05).

APX activity also differed between the genotypes. In L_T_, APX activity was significantly higher in the treated samples after 24 h (*p* < 0.05) and 7 d (*p* < 0.01) of waterlogging treatment, while, in L_S_, it was higher than the control values throughout the experiment, and it increased with the treatment duration (Figure 4A). However, only the results after 7 d for L_S_ were statistically significant (*p* < 0.01).

Considering POD activity (Figure 4B), it was higher in the treated plants after 24 h (*p* < 0.01) and 72 h (*p* < 0.05) in L_T_, and then the activity dropped below the control value at 7 d. In L_S_, POD activity was higher compared to the control (*p* < 0.05) after 72 h when it reached its peak in the treated samples and after 7 d when it was still significantly increased (*p* < 0.01) (Figure 4B).

### 3.3. Assessment of Waterlogging Tolerance Through Gene Expression Analysis

Gene expression analysis was performed by real-time PCR analysis. The comparisons of expression profiles of the selected genes were made between the control and treatment samples of both genotypes at all three time points. Genes selected for this analysis were *Zmpdc1*, *Zmpdc3*, *XET*, *ATPS*, *psbI*, and *psbE*.

**Figure 5:**
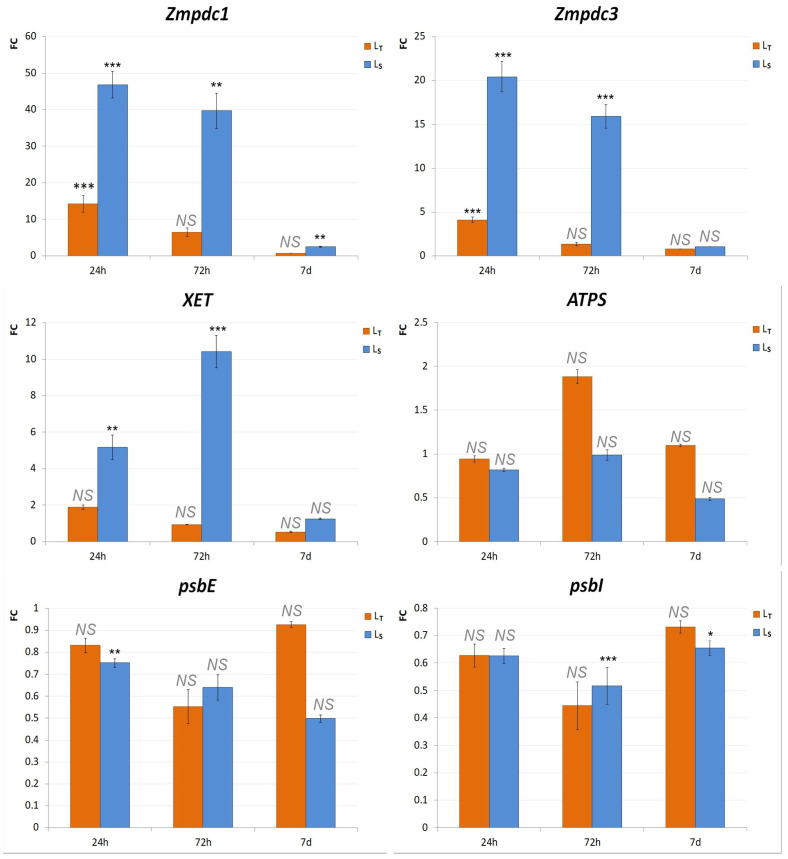
Gene expression analysis of selected genes using real time-PCR. Real-time PCR analysis was performed on selected genes (*Zmpdc1*, *Zmpdc3*, *XET*, *ATPS*, *psbI*, and *psbE)*. Expression patterns are shown as 2^−ΔΔCt^ values (FC) obtained from the ΔΔCt values from the control and treated samples in L_T_ and L_S_ after 24 h, 72 h, and 7 d. L_T_ samples are shown in orange, and L_S_ in blue. The significance of the difference between the control and treatment of each gene was determined by *t*-test and is shown as *** (*p* < 0.001), ** (*p* < 0.01), *(*p* < 0.05), and NS (statistically not significant at *p* > 0.05).

*ATPS* was found not to be differentially expressed under waterlogging conditions in either of the genotypes: there were no statistically significant differences between the control and treatment at any of the time points. Additionally, *XET*, *psbI*, and *psbE* genes showed no significant fold changes in L_T_. The only significant differences were found in *Zmpdc1* and *Zmpdc3* expression after 24 h, where they were both upregulated (FCZmpdc1 = 14.2, FCZmpdc3 = 4.1) in the treated seedlings of L_T_–FCZmpdc1 = 14.2 and FCZmpdc3 = 4.1 (Figure 5). On the other hand, several genes were found to be significantly differentially expressed between the control and treatment samples of L_S_. *Zmpdc1*, *Zmpdc3*, and *XET* were upregulated after 24 h and 72 h. *Zmpdc1* was also upregulated (FCZmpdc1 = 2.5) after 7 d. Additionally, several genes were downregulated, including *psbE* after 24 h and *psbI* after 72 h (Figure 5).

## 4. Discussion

As early sowing is one of the main strategies in increasing crop adaptation to climate change and securing yields necessary to guarantee future food security, better understanding of environmental factors, and their effect on young maize seedlings becomes unavoidable. The earlier sowing of maize in temperate areas is challenged by exposure to suboptimal temperatures and frequent extreme precipitations that lead to waterlogging stress. Herein, two maize genotypes, previously tested for low temperature tolerance on the morphological and molecular levels—via whole transcriptome sequencing [24]—were also evaluated for waterlogging tolerance, using morphological, physiological, and molecular parameters.

Waterlogging has a strong impact on seedling morphological traits, as it significantly reduces root volume, length, and length density [12,33]. Root morphology is a prevalent factor for nutrient and water absorption and for securing a high photosynthesis rate and high grain yield [12,34]. Also, the inhibition of root growth in anaerobic conditions leads to the limitations of shoot growth and reductions in plant height [11]. The waterlogging tolerance of the two genotypes was first tested on root- and shoot-related morphological parameters after variable exposure duration to the stress—24 h, 72 h, and 7 d. It was shown that while there was no significant difference between the genotypes after 24 h, genotype tolerant to low temperatures, L_T_, seemed to be more tolerant to waterlogging conditions after prolonged exposure. In particular, although waterlogging had a negative impact on all analyzed traits in both genotypes, the changes were of a higher magnitude in Ls. Similar results were obtained in several studies in which root length was positively correlated with waterlogging tolerance in maize inbred lines [6,35]. Waterlogging also results in a decrease in the root/shoot ratio [36,37]. Interestingly, despite the reductions in RL, SL, DRW, and DSW, there was an increase in the root/shoot ratio in both genotypes. A possible explanation could be that the shoots are deteriorating at a faster pace than the roots, resulting in skewed results.

Oxygen deficiency caused by waterlogging stress leads to the production of ROS and affects cell membrane stability [38]. Under waterlogging, ROS is necessary for aerenchyma formation, an important adaptive trait which enables oxygen transport from aerobic shoots to anaerobic roots [39]. However, ROS accumulation also causes oxidative damage to the cells [40,41], meaning that a certain balance between ROS generation and scavenging is important for the plants’ survival under waterlogging conditions. The level of oxidative damage can be estimated through assessing lipid peroxidation and antioxidative enzyme activities. Herein, APX and POD were analyzed as it is known that they play a role in cell wall loosening and re-organization, as needed for the formation of aerenchyma. Additionally, mass spectroscopy revealed that isoforms of class III peroxidases and ascorbate peroxidases are involved in waterlogging stress response [42].

The content of MDA is used as an indicator of the lipid peroxidation of cell membranes, which is higher in stress-sensitive genotypes. Also, the performance of plants in response to waterlogging depends closely on plant growth stage, depth of water level, and duration of waterlogging [11]. In our work, the MDA content during treatment differed between the two genotypes and also showed dependence on stress duration, indicating different levels of cell membrane stability caused by time exposure of the stress. In this context, cell membranes were less affected by oxidative stress after 24 h and 72 h in L_T_ and after 7 d in Ls. Higher membrane stability during the first three days of waterlogging stress indicates a higher probability of adapting and surviving under unfavorable conditions. A higher content of MDA in the sensitive maize genotypes under waterlogging conditions was also shown in field experiments in which two water regimes were used—soil moisture of 70–85% field capacity as moderate control and 2–3 cm water layer for six days above the soil surface at the V6 stage as waterlogging stress [10]. Besides waterlogging, MDA was shown to increase in sensitive genotypes and stay unchanged in tolerant ones under different abiotic conditions [43].

The performance of the analyzed peroxidases reflected different patterns depending on the genotype and stress duration. Both APX and POD activities were significantly increased in treated plants after 24 h stress in L_T_, while no significant changes were found at this time point for Ls. Further on, APX and POD displayed different activity directions in L_T_—a decrease and increase after 72 h and vice versa after 7 d, respectively. A completely different pattern was observed in L_S_, in which both enzymes were increased at these two time points. During aerenchyma formation, root cortical cell death is accompanied by ROS production, which might be the result of ROS-producing enzyme gene upregulation and downregulation of those encoding for ROS-scavenging enzymes [39]. It is possible that the differences in POD and APX activities, accompanied by changes in the MDA content at different time points of stress duration could be linked to differences in the induction of aerenchyma formation between the two genotypes.

The waterlogging response was also analyzed on the molecular level by determining the expression patterns of several genes using real-time PCR. Zmpdc1, Zmpdc3, and *XET* genes were specifically chosen for their documented roles in response to anaerobic conditions [27,28]. Conversely, other genes were selected based on their earlier identification as part of the abiotic stress response in the maize genotypes utilized in this research: *ATPS*, *psbI*, and *psbE* [29].

Pyruvate decarboxylase (PDC) is a key enzyme in the ethanol fermentation pathway, an anaerobic respiration pathway activated in hypoxic or anoxic conditions during waterlogging [44]. pdc gene upregulation under waterlogging conditions was shown in various plant species [9,45,46], including maize [43]. According to [27], the expression of Zmpdc1 and Zmpdc3 reached its maximum 24 h after the commencement of the waterlogging treatment, after which it exhibited a decrease. These results corroborate the findings presented in the current research. In both genotypes, the expression levels of Zmpdc1 and Zmpdc3 peaked at 24 h, and it was markedly higher in Ls for both genes. However, no significant difference was found for these two genes after 72 h and 7 d in L_T_, which was not the case in L_S_.

Another enzyme important for waterlogging response is xyloglucan endotransglycosylase (*XET*). This enzyme is active during the final stages of aerenchyma formation, which involves cell wall loosening and degradation [28]. *XET* was previously shown to be involved in response to oxygen deprivation in maize [47] and other plant species [48,49,50]. Interestingly, in this study, there was a significant increase in *XET* gene expression only in the sensitive genotype, L_S_, after 24 h and 72 h. After 7 d, the expression levels were much lower in the treated L_S_ seedlings, nearly the same as the control values. L_T_ showed no significant difference in *XET* expression at any of the time points. As the results of the survival rate, the morphological and physiological parameters indicate that L_T_ could be better equipped to handle anaerobic conditions compared to Ls, and the lack of *XET* upregulation was surprising. The induction of aerenchyma formation takes 24–72 h after the start of anaerobic treatment [28,51], and it could be assumed that aerenchyma formation was delayed in L_T_ compared to Ls. A possible explanation could also be the treatment duration. In many of the research works in which the *XET* upregulation was noted in tolerant genotypes, gene expression analyses were conducted after longer waterlogging treatments—9 to 15 days [52,53].

None of the genes (*XET*, *ATPS*, *psbI*, and *psbE*), previously identified to be included in the abiotic stress response in the analyzed genotypes [28,29], were differentially expressed in L_T_. In L_S_, *psbE* was downregulated after 24 h, and *psbI* expression was decreased after 72 h and 7 d. The psb genes encode proteins that make up the core complex of photosystem II (PSII) and are responsible for its assembly and stability [52]. Anaerobic conditions caused by waterlogging lead to root respiration inhibition, which further affects the rate of photosynthesis and CO_2_ assimilation [8,53]. The downregulation of genes necessary for proper PSII functioning confirmed that waterlogging negatively impacted photosynthesis. The occurrence of psb gene downregulation in L_S_, but not in L_T_, further substantiates the superior performance of the L_T_ genotype under waterlogging conditions.

## 5. Conclusions

The presented results indicate that the genotype identified as tolerant to low temperatures also exhibits a higher degree of tolerance to waterlogging conditions. First, it showed negative changes caused by waterlogging on the root, and shot traits were of lower magnitude in L_T_. Second, higher cell membrane stability at the beginning of the stress in L_T_ indicates a higher probability of adapting to unfavorable conditions. Third, increased activity of both peroxidases indicates the earlier induction of aerenchyma formation in L_T_. Finally, the unchanged expression of *psbE* and *psbI* genes under the stress indicates higher stability of photosystem II. These observations imply a possible overlap in the underlying mechanisms that govern the plant’s responses to these two abiotic stress conditions. It raises intriguing questions about how the plant may utilize similar physiological or biochemical pathways to cope with both cold stress and waterlogging, suggesting a more integrated approach using different -omics (genomics, transcriptomics, proteomics, metabolomics, and phenomics) under both individual and combined stress conditions. This will enable a profound understanding of plant resilience in the face of multiple environmental challenges.

## Figures and Tables

**Table 1:** Sequences of primers for the genes that were subjected to testing.

Gene	ID Primer	Sequence
Pyruvate Decarboxylase1 (Pdc1)	Zmpdc1	F: 5′-CAACTGC TGGACCA TGAAG-3′R: 5′-GCTCGTG TCGTCCTT GTG-3′
Pyruvate Decarboxylase 3 (Pdc3)	Zmpdc3	F: 5′-GAGCTTC CAGGTTA CAGCAC-3′R: 5′-GATTTCCA CCTCAAT CGTGTAC-3′
Xyloglucan endotransglucosylase	XET	F: 5′-ACTACTGCGACGACCGCAAG-3′R: 5′-CCA ACA ATC AGC CCG GTT TT-3′
Chloroplast ATP-sulfurylase	ATPS	F: 5′-ACGTTTGATGTGCTCCAGTG-3′R: 5′-CTTAGCAAGCGGTTCTTTGC-3′
Photosystem II cytochrome b559 alpha subunit	psbE	F: 5′-TCGGTTTTCTACTGGCTGCT-3′R: 5′-ACCCACGAATCTCAATGACC-3′
Photosystem II reaction center protein I	psbI	F: 5′-AGTGGATCCGTGTGGTAAGG-3′R: 5′-CAGGATTACGTCCTGGGTCA-3′

## Data Availability

Data are contained within the article.
